# Coexisting primary tumors from esophageal cancer and myelodysplastic syndromes: A case report

**DOI:** 10.1002/ccr3.4872

**Published:** 2021-09-22

**Authors:** Hiroki Imamura, Shigeyuki Tamura, Hideki Hattori, Shinya Kidogami, Yukako Mokutani, Tomoya Kishimoto, Yasuji Hashimoto, Hajime Hirose, Shinichi Yoshioka, Maki Kuwayama, Shunji Endo, Yo Sasaki

**Affiliations:** ^1^ Department of Surgery Yao Municipal Hospital Yao Japan; ^2^ Department of Hematology Yao Municipal Hospital Yao Japan

**Keywords:** double primary cancer, esophageal cancer, myelodysplastic syndromes, without surgery

## Abstract

This is the first report of the double primary cancer of esophageal cancer (EC) and myelodysplastic syndromes (MDS) treated without esophagectomy. Previously reported cases of the double cancer mostly describe secondary MDS arising after treatment for EC. The double primary cancer was manageable with close follow‐ups for possible recurrence.

## Graphical abstract

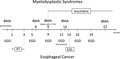

## 1 INTRODUCTION

This is the first report of the double primary cancer of esophageal cancer (EC) and myelodysplastic syndromes (MDS) treated without esophagectomy. Previously reported cases of the double cancer mostly describe secondary MDS arising after treatment for EC. The double primary cancer was manageable with close follow‐ups for possible recurrence.

In recent years, due to developments in medical examinations, simultaneous double cancer (SDC) has been more often detected than before. Treatment for SDC is challenging due to the simultaneity, but it can be even more challenging when a patient has comorbidities that limit treatment options.

Simultaneous double cancer involving EC and MDS is relatively a rare finding with several case reports available hitherto. However, these reports almost always describe secondary MDS, which arises as an adverse effect of treatment for EC. On the contrary, SDC with EC and primary MDS is extremely rare with only one article published before.^1^ Moreover, no one has ever reported a case of the two concurrent primary neoplasms treated without esophagectomy.

## 2 CASE HISTORY

**FIGURE 1:**
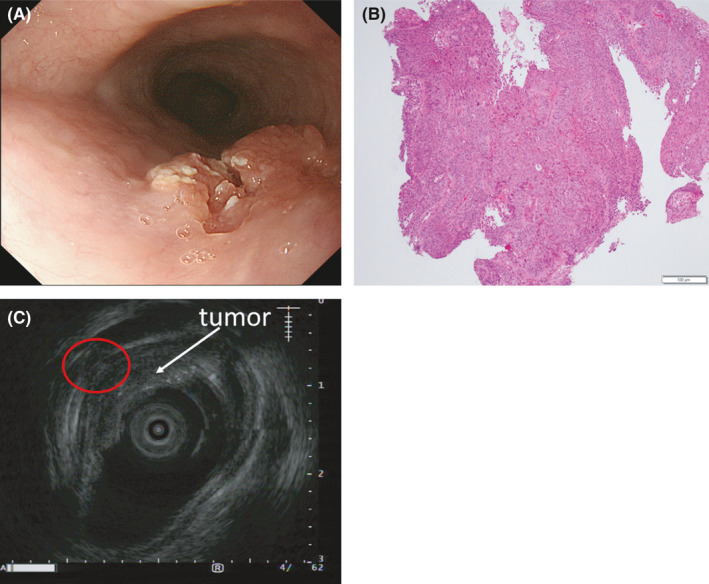
Esophageal findings at presentation (A) Esophagogastroduodenoscopy (EGD) reveals esophageal carcinoma 23 cm from the front teeth (B) A pathological picture of esophageal squamous cell carcinoma (C) Endoscopic ultrasound shows tumor invasion into the submucosal layer (red circle)

**FIGURE 2:**
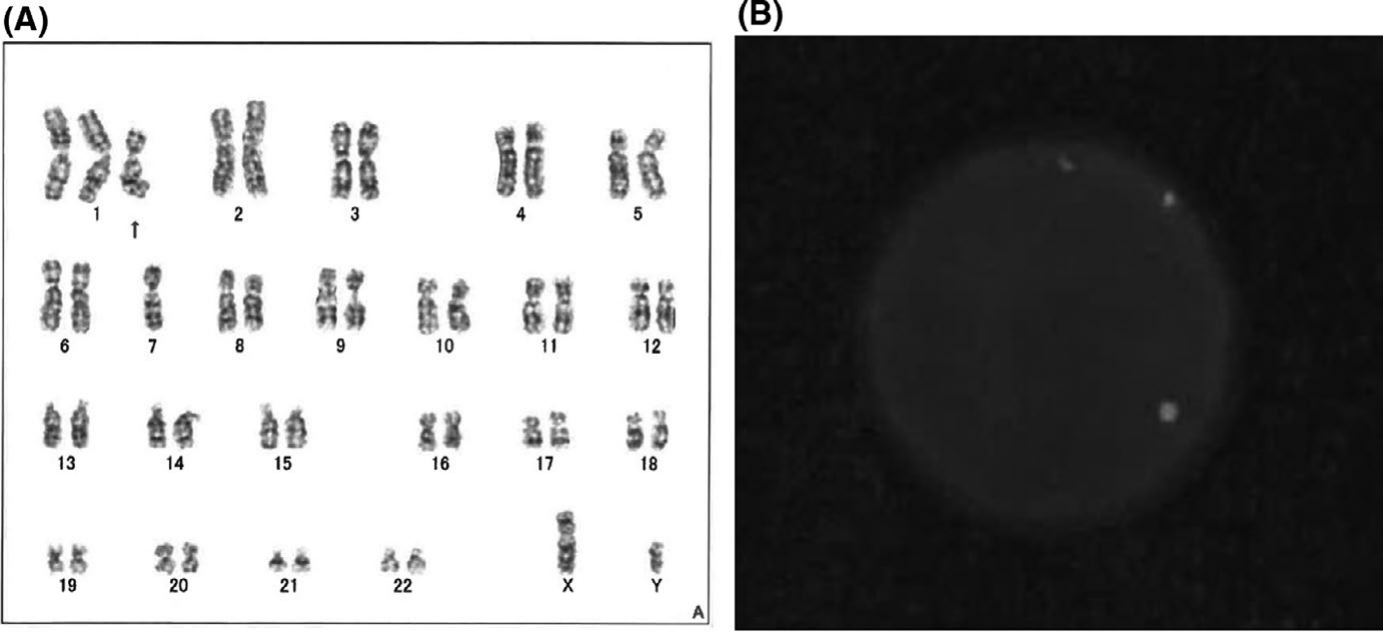
Bone marrow findings at presentation (A) Bone marrow aspiration samples reveal chromosomal abnormality of der(1;7) (q10;p10) in 8 out of 10 cells (B) Fluorescence in situ hybridization shows only one isolated red signal for 7q31 and two green signals for D7Z1(7cen) demonstrating the presence of a 7q31 deletion

**FIGURE 3:**
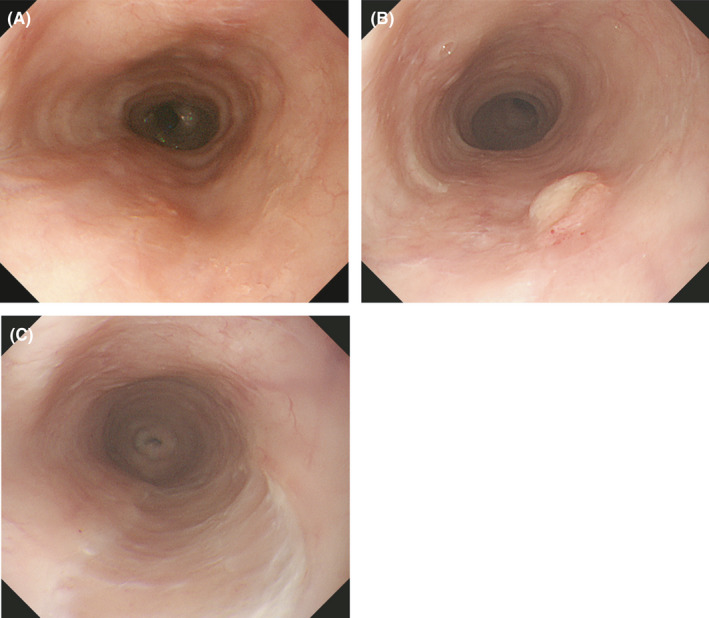
Endoscopic findings after treatment (A) After radiation monotherapy, complete disappearance of the esophageal tumor is achieved (B) EGD 6 months after radiation shows local recurrence of the tumor (C) EGD performed 9 months after ESD showed complete disappearance of the tumor

A 68‐year‐old man was referred to our hospital for anemia of unknown cause. His past medical history was notable for multiple surgeries for thoracic and abdominal aortic dissection, chronic kidney disease (CKD), arterial aneurysm in subclavian artery, and hypertension for which he takes amlodipine. Physical examinations at presentation showed pallor conjunctiva. He was an ex‐smoker and never drank alcohol. Laboratory data showed a slight thrombocytopenia (112,000/µl), normocytic anemia (hemoglobin (Hb): 8.6 g/dl, reticulocytes: 14.6%, mean corpuscular volume: 80 Fl, and serum iron: 28 µg/dl), elevated creatinine level (1.41 mg/dl), and elevated erythropoietin (EPO) level (589 U/ml). His hemoglobin level was 14.0 g/dl 7 months before presentation. A gastrointestinal tumor was suspected. Esophagogastroduodenoscopy (EGD) and colonoscopy were performed, revealing an esophageal tumor 23 cm from the front teeth (Figure 1a). Pathological findings were compatible with esophageal squamous cell carcinoma (Figure 1b). Endoscopic ultrasound showed tumor invasion into the submucosal layer (Figure 1c). Contrast‐enhanced computed tomography (CT) showed no lymphadenopathy or metastatic lesions (cT1bN0M0, cStage I, Union for International Cancer Control (UICC), 8th edition^2^). However, the esophageal tumor was unlikely to be the cause of the observed amount of bleeding. Considering several findings in peripheral blood including the absence of bilirubin elevation and the presence of enough reticulocytes and elevated EPO level, some types of anemia were unlikely such as hemolytic anemia, aplastic anemia, and renal anemia. We consulted a hematologist, and bone marrow aspiration was conducted to exclude hematologic diseases, revealing slight erythroid dysplasia. According to the WHO classification,^3^ MDS‐single lineage dysplasia was diagnosed with a poor prognostic chromosomal abnormality of der(1;7; q10;p10), including the presence of a 7q31 deletion (Figure 2). The diagnosis of the double primary EC and MDS was made. The MDS of this patient was classified as intermediate risk by the revised international prognostic scoring system (IPSS‐R),^4^ suggesting that the estimated prognosis was 3 years. In the treatment for EC with submucosal invasion, radiation monotherapy was selected because neither surgery nor chemoradiotherapy was assumed to be indicated due to the patient's complicated history of surgery for thoraco‐abdominal aortic dissection, CKD, and MDS. Radiation therapy at 59.4 Gy/33 fr was initiated, resulting in the complete disappearance of the tumor on EGD (Figure 3a). The patient was followed up by regular EGD 2, 6, and 9 months after radiation therapy as well as follow‐up CTs every 4 months, and local recurrence was observed at 9 months (Figure 3b). Since the relapsed lesion was recognized as submucosal invasion into the middle one‐third (sm2) and no other types of recurrence (eg, distant metastases) were detected, endoscopic submucosal dissection (ESD) was performed. Pathological findings showed that the resected lesion was a squamous cell carcinoma with sm2 invasion, and the resected margin was negative. The relapsed cancer has been completely resected, and no local recurrence has been observed for 9 months since ESD (Figure 3c) without any lymph node recurrence or distant metastasis.

**FIGURE 4:**
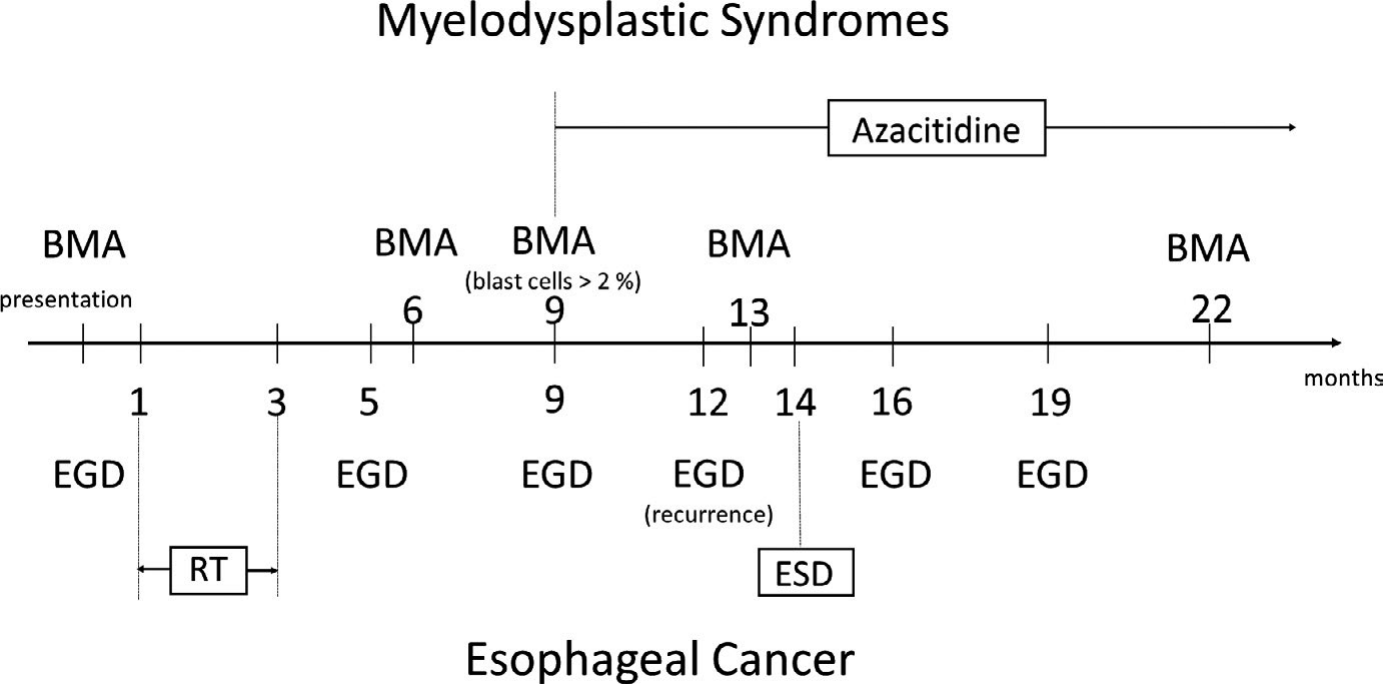
The whole clinical course of both diseases. Abbreviations: BMA, bone marrow aspiration; EGD, esophagogastroduodenoscopy; RT, radiation therapy; and ESD: endoscopic submucosal dissection

For the treatment of the MDS, we first chose supportive therapy with methenolone and he was subject to bone marrow aspiration follow‐up, because his hemoglobin level was stable. However, 7 months after presentation, the anemia had gradually progressed (Hb: 6.1 g/dl), and exacerbation of dysplasia with an elevation of blast cells of 2 or more percent was observed on the bone marrow aspiration. The MDS was classified into the high‐risk group at this point. Initiation of chemotherapy was warranted, and azacitidine was administered and has continued for 11 months. The MDS is now well controlled and shows no progression under azacitidine. Complete remission was achieved with the disappearance of der(1;7; q10;p10) and −7q. The whole clinical course of the two diseases is summarized in Figure 4.

## 3 DISCUSSION

Esophageal cancer and MDS are both known for their high mortality. The statistics from Japan report 11,543 deaths from EC in 2013, which accounts for 3.2% of all deaths from malignant neoplasms.^5^ MDS is another progressive disease that yields a poor prognosis. Xiaomei et al.^6^ reported that the 3‐year survival rate is only 35% in the American population. Although some case reports refer to double cancer of EC and MDS, we must pay attention to the fact that almost all these reports describe therapy‐related (ie, secondary) MDS, not primary MDS. As far as we searched, except for several reports orally presented at local conferences, the only published case of double primary EC and MDS was presented by Okumura et al,^1^ who reported four cases of the double cancer treated with esophagectomy. In treating a patient with double primary cancer of EC and MDS, an elaborate treatment strategy is required because EC limits the treatment options for MDS, and vice versa. The prognosis of EC is assessed by TNM classification, and the prognosis of MDS is commonly assessed by the International Prognostic Scoring System (IPSS),^7^ or by IPSS‐R. The estimated prognosis of each disease is important in establishing a treatment strategy for the concurrent disease of EC and MDS. Although there is limited evidence due to the rarity of the disease, we assume that, in patients with concurrent EC and MDS, esophagectomy can be an indication when patients can tolerate surgery and have low‐risk MDS (eg, less than three points with IPSS‐R). On the contrary, for patients who are intolerant of surgery or suffer from high‐risk MDS (eg, more than 4.5 points with IPSS‐R), chemoradiotherapy or radiation monotherapy is indicated for EC. According to Japanese statistics, stage I EC has a 5‐year overall survival rate of 76.8%.^5^ On the contrary, the MDS of this patient scored four points on the IPSS‐R, suggesting that the patient should be classified into the intermediate‐risk group, which has a 3‐year overall survival.^4^ According to the Japanese guidelines, stage I EC is usually treated with surgery if the patient can tolerate it or with chemoradiotherapy or radiotherapy alone if otherwise.^8^ However, the evidence has not been established regarding the tolerance to surgery for EC in patients with concurrent MDS.

Okumura et al.^1^ reported that surgery for EC was a treatment option in patients with concurrent MDS. However, considering that our patient had multiple medical history including thoraco‐abdominal surgery for aortic dissection and CKD in addition to his old age, esophagectomy was judged to be intolerable. According to the Japanese guidelines, definitive chemoradiotherapy or radiotherapy is a treatment of choice for patients who cannot tolerate surgery.^5^ Chemoradiotherapy has a higher therapeutic response than radiotherapy alone and thus is recommended over radiotherapy. However, it is also described in the Japanese guidelines that radiotherapy may be selected when chemoradiotherapy is precluded by factors such as advanced age, presence of complications.^8^ In addition to the complicated medical history and old age, our patient carried a great risk because his MDS was accompanied by chromosomal abnormality of 7q31 deletion, which is a known predisposing factor for myeloid transformation. Moreover, chemotherapeutic agents causing bone marrow suppression were not considered an indication due to coexisting MDS. Since cisplatin, which is commonly used as a chemotherapeutic agent for EC, is known to cause therapy‐related MDS,^9^ it could exacerbate MDS of our patient. Considering all these issues, chemotherapy would have provided harm rather than benefit to our patient, and thus, radiation monotherapy was selected. Radiation therapy is reported to be associated with a higher rate of local recurrence than chemoradiotherapy,^10^ and recurrence occurred in our patient. According to the Japanese guidelines, as long as recurrent lesion is localized, salvage treatment such as surgery and endoscopic resection may be adopted for local recurrence.^8^ Due to the development in endoscopic techniques, ESD is often reported to be a reasonable treatment with high effectiveness and minimal invasiveness.^11^

Treatment for MDS is sometimes not necessary when a patient is asymptomatic.^12^ It has also been reported that patients with intermediate‐risk MDS according to the IPSS‐R are indicated for treatment both for low‐ or very‐low‐risk MDS and for high‐ or very‐high‐risk MDS according to the patients' condition.^4^ Watchful observation with regular bone marrow aspiration seemed to be a reasonable option for our patient because of his coexisting EC. The patient's condition was stable without deterioration of anemia for 9 months after presentation, when bone marrow aspiration showed at least 2% blast cells. MDS was then classified as high risk according to IPSS‐R. Considering the poor prognostic abnormality, a leukemic change from MDS was highly likely to occur, indicating that therapeutic intervention was necessary. Intensive therapy, such as allogeneic hematopoietic stem cell transplantation, was not indicated because of his EC and old age. We chose lower intensity therapy, with a hypomethylation agent (azacitidine). After initiation of azacitidine, his anemia did not progress any further, and his blast cell count returned to a normal level. The patient remains in a stable condition 11 months after the initiation of azacitidine.

In conclusion, simultaneous double primary tumors of EC and MDS were manageable if we can assess the risks and benefits of each treatment alternative available to us even when surgery was not indicated due to the patient's comorbidities.

## CONFLICTS OF INTEREST

The authors declare that we have no competing interests.

## AUTHOR CONTRIBUTIONS

HI and ST treated EC throughout the clinical course. HH and MK treated MDS. All the authors made substantial contributions to conception and design, were involved in drafting and revising the manuscript for important intellectual content, and approved of the final version of the manuscript to be publication. All the authors agreed to be accountable for all aspects of the work in ensuring that questions related to the accuracy or integrity of any part of the work are appropriately investigated and resolved.

## 4 ETHICAL APPROVAL

Ethics approval was obtained from Ethics Committee of Yao Municipal Hospital with approval number of YMH122320‐102. This manuscript was written conforming to Declaration of Helsinki.

## CONSENT

Consent for publication of this manuscript was obtained from the patient.

## Data Availability

The data that support the findings of this study are available from the corresponding author upon reasonable request.
